# Linkage and related analyses of Barrett's esophagus and its associated adenocarcinomas

**DOI:** 10.1002/mgg3.211

**Published:** 2016-03-14

**Authors:** Xiangqing Sun, Robert Elston, Gary W. Falk, William M. Grady, Ashley Faulx, Sumeet K. Mittal, Marcia I. Canto, Nicholas J. Shaheen, Jean S. Wang, Prasad G. Iyer, Julian A. Abrams, Joseph E. Willis, Kishore Guda, Sanford Markowitz, Jill S. Barnholtz‐Sloan, Apoorva Chandar, Wendy Brock, Amitabh Chak

**Affiliations:** ^1^ Department of Epidemiology and BiostatisticsCase Western Reserve UniversityClevelandOhio; ^2^ Case Comprehensive Cancer CenterCase Western Reserve University School of MedicineClevelandOhio; ^3^ University of Pennsylvania Perelman School of MedicinePhiladelphiaPennsylvania; ^4^ Clinical Research DivisionFred Hutchinson Cancer Research CenterSeattleWashington; ^5^ Gastroenterology DivisionUniversity of Washington School of MedicineSeattleWashington; ^6^ Division of Gastroenterology and HepatologyUniversity Hospitals Case Medical CenterCase Western Reserve University School of MedicineClevelandOhio; ^7^ Division of Gastroenterology and HepatologyLouis Stokes Veterans Administration Medical CenterCase Western Reserve University School of MedicineClevelandOhio; ^8^ Department of SurgeryCreighton University School of MedicineOmahaNebraska; ^9^ Division of GastroenterologyJohns Hopkins Medical InstitutionsBaltimoreMaryland; ^10^ Center for Esophageal Diseases & SwallowingUniversity of North Carolina at Chapel Hill School of MedicineChapel HillNorth Carolina; ^11^ Division of GastroenterologyWashington University School of MedicineSt. LouisMissouri; ^12^ Division of Gastroenterology and HepatologyMayo ClinicRochesterMinnesota; ^13^ Department of MedicineColumbia University Medical CenterNew YorkNew York; ^14^ Department of PathologyUniversity Hospitals Case Medical CenterCase Western Reserve University School of MedicineClevelandOhio; ^15^ Division of General Medical Sciences (Oncology)Case Comprehensive Cancer CenterClevelandOhio; ^16^ Department of Medicine and Case Comprehensive Cancer CenterCase Medical CenterCase Western Reserve UniversityClevelandOhio

**Keywords:** Barrett's esophagus, esophageal adenocarcinoma, familial, genetics, linkage

## Abstract

**Background:**

Familial aggregation and segregation analysis studies have provided evidence of a genetic basis for esophageal adenocarcinoma (EAC) and its premalignant precursor, Barrett's esophagus (BE). We aim to demonstrate the utility of linkage analysis to identify the genomic regions that might contain the genetic variants that predispose individuals to this complex trait (BE and EAC).

**Methods:**

We genotyped 144 individuals in 42 multiplex pedigrees chosen from 1000 singly ascertained BE/EAC pedigrees, and performed both model‐based and model‐free linkage analyses, using S.A.G.E. and other software. Segregation models were fitted, from the data on both the 42 pedigrees and the 1000 pedigrees, to determine parameters for performing model‐based linkage analysis. Model‐based and model‐free linkage analyses were conducted in two sets of pedigrees: the 42 pedigrees and a subset of 18 pedigrees with female affected members that are expected to be more genetically homogeneous. Genome‐wide associations were also tested in these families.

**Results:**

Linkage analyses on the 42 pedigrees identified several regions consistently suggestive of linkage by different linkage analysis methods on chromosomes 2q31, 12q23, and 4p14. A linkage on 15q26 is the only consistent linkage region identified in the 18 female‐affected pedigrees, in which the linkage signal is higher than in the 42 pedigrees. Other tentative linkage signals are also reported.

**Conclusion:**

Our linkage study of BE/EAC pedigrees identified linkage regions on chromosomes 2, 4, 12, and 15, with some reported associations located within our linkage peaks. Our linkage results can help prioritize association tests to delineate the genetic determinants underlying susceptibility to BE and EAC.

## Introduction

National statistics estimate 18,140 new cases of esophageal cancer, the majority adenocarcinomas, in 2014 (Siegel et al. 2014). The incidence of esophageal adenocarcinoma (EAC) in the United States has increased dramatically in the past four decades, especially in white males (Blot et al. 1991; Pera et al. 1993; Devesa et al. 1998; Pohl and Welch 2005; Siegel et al. 2014). The prognosis remains poor, with a 5‐year survival below 20% (Siegel et al. 2014). Almost all EACs originate in Barrett's epithelium, a premalignant condition in which normal stratified squamous epithelium is replaced by metaplastic specialized intestinal type columnar epithelium (Haggitt et al. 1978; Hameeteman et al. 1989; Reid et al. 1992; Cameron et al. 1995; Hirota et al. 1999; Ruol et al. 2000; Spechler 2002; Sharma et al. 2004). We, and others, originally recognized that Barrett's esophagus (BE) and EAC aggregate in a proportion of families (Crabb et al. 1985; Prior and Whorwell 1986; Jochem et al. 1992; Eng et al. 1993; Fahmy and King 1993; Poynton et al. 1996; Chak et al. 2002, 2006). Because BE and EAC are epidemiologically similar and there is evidence that nearly all EACs arise in Barrett's epithelium, we have considered BE and EAC to be part of a single binary trait termed familial Barrett's esophagus (FBE) (Haggitt et al. 1978; Cameron et al. 1995; Hirota et al. 1999; Ruol et al. 2000).

A few linkage analyses of BE, EAC, or gastroesophageal reflux disease (GERD) have been published, but they are in relatively small to moderate‐sized samples. Hu et al. (2000) found linkage evidence of pediatric GERD for a locus on chromosome 13q14 in five families, but Orenstein et al. (2001, 2002) excluded linkage at this locus in a linkage study of six infantile GERD families. Orloff et al. (2011) studied BE/EAC in 31 sib pairs (21 concordant‐affected and 11 discordant sibling pairs) by model‐free linkage, and reported linkage to three genes *MSR1* (8p), *ASCC1* (10q), and *CTHRC1* (8q). Our initial studies of FBE determined that families with three or more affected members develop esophageal cancer at an earlier age compared to families with only one or two affected members, suggesting a genetic basis for this complex trait (Chak et al. 2006, 2009). Furthermore, segregation analysis of singly ascertained families provided evidence against a sporadic environmental model and supported a genetic basis for FBE (Sun et al. 2010). The results of the segregation analysis led us to conduct a linkage study in 42 pedigrees to identify genomic regions that might contain genetic variants that predispose individuals to develop BE and EAC.

## Methods

### Data

#### Pedigree accrual and trait definition

The multi‐center methodology for approaching probands and accruing FBE pedigrees has been previously described (Chak et al. 2006; Sun et al. 2010). Recruitment occurred at eight hospitals during variable periods over the past 9 years. The FBE study was approved by the institutional review board for human investigation at each participating hospital and registered on clinicaltrials.gov (NCT00288119).

A questionnaire that collects data on relevant covariates is administered to affected probands and all family members who consent to participate. A diagnosis of BE or EAC is confirmed by review of endoscopy and pathology records, and is defined as affected in our study (Chak et al. 2002, 2006). This definition of the affected agrees with a report that BE and EAC have high genetic correlation (*r*_g_ = 1.0) (Ek et al. 2013). The definition of the EAC phenotype requires the presence of adenocarcinoma on biopsy taken from a mass that predominantly involves the tubular esophagus, and the definition of BE requires the endoscopic appearance of columnar mucosa in the tubular esophagus with a biopsy from that area demonstrating intestinal metaplasia. Biopsies showing intestinal metaplasia from an irregular Z line or the gastroesophageal junction are not considered part of the trait. Individuals without a history of BE or EAC are defined as unaffected.

We thus obtained a set of data with 1000 singly ascertained Barrett's esophagus pedigrees comprising 10,594 individuals that was used to estimate the genetic mode of inheritance of FBE. The dataset used here is a corrected and expanded version of the data that were analyzed by Sun et al. (2010). Clinical covariate data are missing from family members who declined participation, family members who did not complete the clinical questionnaire, and those who are deceased. From these, 42 informative multiplex pedigrees were chosen that comprise in total 1132 individuals with disease status available, and 144 members with blood samples available were genotyped using the Illumina GoldenGate Human Linkage V Panel. There were 5687 autosomal SNPs genotyped. Although 37 of them have a missing rate >0.05 (which were not located in the linkage regions that we identified), because their missing genotypes could be inferred from their relatives this was done, and they were not excluded from the linkage analyses. The sample call rates are >0.95 for all samples, so all the samples were used in the analysis. Relationship and Mendelian inconsistencies were checked using the genome‐wide SNPs with the programs RELTEST and MARKERINFO in the S.A.G.E. package (http://darwin.cwru.edu/sage/). Fourteen relative pairs that were identified as unrelated and two full sib pairs identified as half sibs were accordingly corrected. There were 395 SNPs that have Mendelian inconsistencies and they were automatically excluded from the analyses. After relationship correction, the genotyped pedigrees include 78 affected and 66 unaffected individuals, comprising 111 sib pairs, 10 half sib pairs, 18 avuncular pairs, and nine cousin pairs.

### Segregation models

To find appropriate models for model‐based linkage analysis, we fitted segregation models using the program SEGREG in S.A.G.E. 6.3. This was done on both the 1000 singly ascertained pedigrees with 10,594 individuals and the 42 linkage informative pedigrees with 1132 individuals. We fitted two types of statistical segregation models: the finite polygenic mixed model (FPMM) (Fernando et al. 1994; Lange 1997) and the multivariate logistic model (MLM) (Karunaratne and Elston 1998), which assume that the genetic locus has two susceptibilities, transmitted in either a dominant or recessive mode of inheritance. In fitting the FPMM model, we included a polygenic component in the model. In fitting the MLM model, we assumed no residual associations between family members because of the theoretical difficulty this entails (Karunaratne and Elston 1998). Two covariates – sex and founder status – are available for all the genotyped individuals in the linkage pedigrees, and for model‐based linkage analysis we included these two variables as covariates of the genotype susceptibilities.

In order to adjust for ascertainment, in fitting the segregation models to the 1000 pedigrees we assumed single ascertainment; when fitting models to the 42 pedigrees, we not only assumed single ascertainment, but also specified a population prevalence constraint, assuming an average population prevalence of 1% for BE/EAC (Ronkainen et al. 2005; Zagari et al. 2008). The rationale for constraining the prevalence is that single ascertainment cannot fully adjust for how the 42 multiplex pedigrees were ascertained, and using a population prevalence constraint in the pedigree likelihood function, instead of the higher prevalence of familial BE in BE patients (Chak et al. 2006), helps better estimate the trait allele frequency in founders of the pedigrees (Sun et al. 2012).

### Linkage analyses

Among the 42 pedigrees containing individuals genotyped for linkage, there were 18 pedigrees that include at least one affected female. Because BE/EAC is less prevalent in females, these 18 pedigrees are expected to be more genetically homogeneous. The following analyses were therefore separately performed on the 42 pedigrees and the subset of 18 pedigrees.

#### Model‐based linkage analyses

Using the dominant and recessive models estimated for the 42 linkage pedigrees, we performed both multipoint and single marker linkage analysis for the autosomal data with the programs MLOD and LODLINK, respectively, of the S.A.G.E. 6.3 package. The SNPs used for the multipoint linkage were thinned to have minor allele frequency (MAF) ≥ 0.2 and the intervals between any two consecutive SNPs at least 0.2 cM. The single marker model‐based linkage analysis was performed for all the SNPs.

#### Model‐free linkage analyses

Successively using the programs FREQ, GENIBD, and SIBPAL in the S.A.G.E. program package, allele frequencies and sibpair identity by descent (IBD) were estimated for all the SNPs and single marker model‐free linkage analysis was performed. By using the W4 option in SIBPAL, the optimally weighted average of the squared sibpair trait sum and squared sibpair trait difference (Shete et al. 2003) was regressed on the sibpair IBD sharing for each SNP. To be comparable to the model‐based linkage, sex was included in the regression model as a binary covariate (sibpairs concordant or discordant for sex). For SNPs with nominal *P* < 0.05, empirical *P*‐values were evaluated by permutation, the number of permutations determined for the *P*‐values to be within 20% of their true values with 95% confidence, up to 100,000 permutations. Because the permutation test currently in SIBPAL can lead to inflated significance of very small *P*‐values, especially in larger sibships (Shete et al. 2003), we devised a more appropriate permutation test (described in the Supplementary materials) that in most cases increased the *P*‐value: this *P*‐value (or equivalent lod) is used here whenever it was found to be larger than the asymptotic *P*‐value.

### Association analysis

Association tests were also performed for each SNP in the linkage panel, separately using the 42 pedigrees and the 18 female‐affected pedigrees. In order to account for familial correlations, this analysis was performed using the program ASSOC in S.A.G.E. The association model in ASSOC can include both a polygenic variance component and a common sibship variance. However, both these two variance components converged to 0 on testing 95% of the SNPs, and only one of them could be estimated for the remaining 5% of the SNPs. In testing the association, each SNP was coded in three ways – dominant, recessive, or additive. Sex and founder status were included as covariates of FBE (i.e., of the logit of FBE). For each SNP, the minimum association *P*‐value among the three tests (additive, dominant or recessive) less than 0.01 by a likelihood ratio test is reported.

## Results

### Segregation models

On fitting the FPMM model with 1 polygenic locus, we found that – on the basis of Akaike's A information criterion (AIC) – using the 1000 pedigrees or the 42 linkage pedigrees, the best‐fitting model was found to be a dominant model (the heterozygous genotype and the homozygous minor allele genotype have higher disease risk); on fitting the MLM model, using either dataset the best‐fitting model was a recessive model (the minor allele homozygous genotype has higher risk) (Table 1). In addition to the mode of transmission, the penetrance probabilities estimated from the 1000 pedigrees are also very close to those from the 42 pedigrees (Table S1); however, the estimated trait locus allele frequencies from the two sets of pedigrees are very different, the one from the 42 linkage pedigrees having a much higher susceptibility allele frequency. This is expected because the 42 linkage pedigrees were ascertained from the 1000 pedigrees for having more affected family members. In view of this, we used both the dominant and recessive models fitted to the 42 linkage pedigrees, allowing for single ascertainment and using a prevalence constraint, for model‐based linkage analyses of these 42 genotyped pedigrees (Table 1, models 1 and 2).

**Table 1:** Segregation models estimated from the 42 linkage pedigrees and the 1000 pedigrees

Parameters (±standard errors)^a^	42 pedigrees^b^		1000 pedigrees^c^	
	1. Dominant (FPMM)	2. Recessive (MLM)	3. Dominant (FPMM)	4. Recessive (MLM)
*β*_AA_	−3.25 ± 0.10	−0.84^d^	−2.26 ± 0.20	−0.63 ± 0.50
*β*_AB_	−3.25 ± 0.10	−35.36^d^	−2.26 ± 0.20	−5.17 ± 0.21
*β*_BB_	−34.40	−35.36^d^	−5.72 ± 0.25	−5.17 ± 0.21
Sex	−2.52 ± 0.08	−2.09 ± 0.53	−1.39 ± 0.20	−1.47 ± 0.24
Founder	−3.22 ± 0.10	−2.69 ± 0.79	−1.61 ± 0.28	−1.70 ± 0.33
Polygenic variance	5.50 ± 0.17		1.06 ± 0.27	
q_A_	0.05 ± 0.01	0.21 ± 0.02	0.005 ± 0.002	0.07 ± 0.02
AIC	573.75	586.85	1559.49	1740.22

a All parameter estimates are on the logit scale except for q_A_, the susceptibility allele; *β* is the logit of susceptibility (probability of ever having disease) for individuals with a given genotype (AA, AB, or BB); sex and founder are two mean‐centered covariates of the (logit of) susceptibility.

b Adjusting for single ascertainment and using a prevalence constraint.

c Adjusting for single ascertainment.

d Flat or near‐flat likelihood in the region of the estimates.

### Linkage analyses

The potential genetic heterogeneity of BE/EAC and relatively small sample size make it difficult to find good evidence of linkage; we therefore performed multiple linkage analyses and summarize here the most consistent results. The regions or positions that are identified by at least two linkage analysis methods, or by one linkage analysis method, but also show some possible evidence of association, are highlighted in the Tables. Full detailed results identified by any of the analyses are reported in the Tables S2 and S3.

#### Linkage analysis of the 42 pedigrees

The regions and locations most suggestive of linkage that were identified in the 42 pedigrees by at least two analyses are seen in Table 2; they are located on chromosomes 2, 4, 8, and 12.

**Table 2:** Linkage (LODLINK, MLOD, SIBPAL) and association (ASSOC) results identified using all 42 pedigrees with evidence of suggestive linkage or association by at least two of the methods

Chr	Position (cM)	Model‐based linkage										Model‐free linkage	Association		
		Recessive						Dominant							
		LODLINK (lod > 2)			MLOD (lod > 2)			LODLINK (lod > 2)	MLOD (lod > 3)			SIBPAL (−log_10_(*P*) > 2.92)	ASSOC (*P* < 0.01)		
		Position	SNP	Lod	Position	SNP	Lod	Lod	Position	SNP	Lod	−log_10_(*P*)	Position	SNP	*P*‐value
2	**174–190 (2q31)**	**174.27**	**rs2032965**	**2.15**	**178.55**	**rs11078**	**2.08**	≤1.72	**177.35**	**rs17664**	**3.13**	≤1.14			
		**180.30**	**rs935866**	**2.30**	**179.18**	**rs907956**	**2.39**		**178.55**	**rs11078**	**3.49**				
					**179.48**	**rs3813817**	**3.22**		**179.18**	**rs907956**	**3.64**				
					**179.76**	**rs2033866**	**3.47**		**179.48**	**rs3813817**	**3.93**				
					**180.3**	**rs935866**	**3.60**		**179.76**	**rs2033866**	**4.02**				
					**181.55**	**rs711814**	**2.60**		**180.3**	**rs935866**	**3.97**				
									**181.55**	**rs711814**	**3.62**				
									**183.55**	**rs1438048**	**3.43**		**189.74**	**rs1569135**	**0.0005**
									**183.87**	**rs1400826**	**3.41**		**190.70**	**rs11894667**	**0.0071**
4	**48–59 (4p14)**						≤0.98	≤1.50	49.04	rs1553923	3.27	≤1.20			
									49.65	rs1392056	3.31				
									51.18	rs902659	3.14				
		**53.25**	**rs13134283**	**2.02**					**53.25**	**rs13134283**	**3.16**				
									56.7	rs1115259	3.38				
									58.87	rs278973	3.34				
									59.46	rs2035383	3.37				
	72–73 (4q13)	73.21	rs1390351	2.31			≤−0.63	≤1.36				≤1.18	72.78	rs11727819	0.0077
8	99–125 (8q22)			≤1.78			≤0.35	≤1.53	100.14	rs11785	3.01	≤1.28			
									101.69	rs2245832	3.09				
									102.23	rs714046	3.08				
									111.38	rs2131858	2.94				
									118.86	rs1628373	3.43				
									119.53	rs769322	3.71				
									121.52	rs2034844	3.83				
									122.14	rs1433396	3.75				
									122.36	rs1021898	3.73				
									124.76	rs755649	3.22		125.32	rs748856	0.0024
12	**110–120 (12q23)**			≤1.83	**111.16**	**rs1492254**	**2.55**	≤1.23	**111.16**	**rs1492254**	**3.35**	≤0.89			≥0.07
					**112**	**rs35723**	**2.57**		**112**	**rs35723**	**3.31**				
					**112.36**	**rs11609259**	**2.54**		**112.36**	**rs11609259**	**3.25**				
					**113.35**	**rs3205421**	**3.26**		**113.35**	**rs3205421**	**3.94**				
					**115.83**	**rs703618**	**2.33**		**114.73**	**rs6539055**	**3.73**				
					**116.17**	**rs1000295**	**2.31**		**115.83**	**rs703618**	**3.83**				
									**116.17**	**rs1000295**	**3.80**				
									**117.17**	**rs746035**	**3.68**				
									**119.05**	**rs1862032**	**3.09**				

Note: The regions or SNPs in bold are those identified by at least two linkage analyses. Regions or SNPs with suggestive linkage (lod > 2, or (−log_10_(*P*) > 2.92) or association (unadjusted *P* < 0.01) are reported; but, for multipoint linkage under the dominant model, only those with lod > 3 are reported because this model produces an overall higher average lod than the other linkage results. If no suggestive signal in a region is identified by an analysis, the largest signal by the analysis at the region is reported.

##### Model‐based linkage analyses

**Figure 1:**
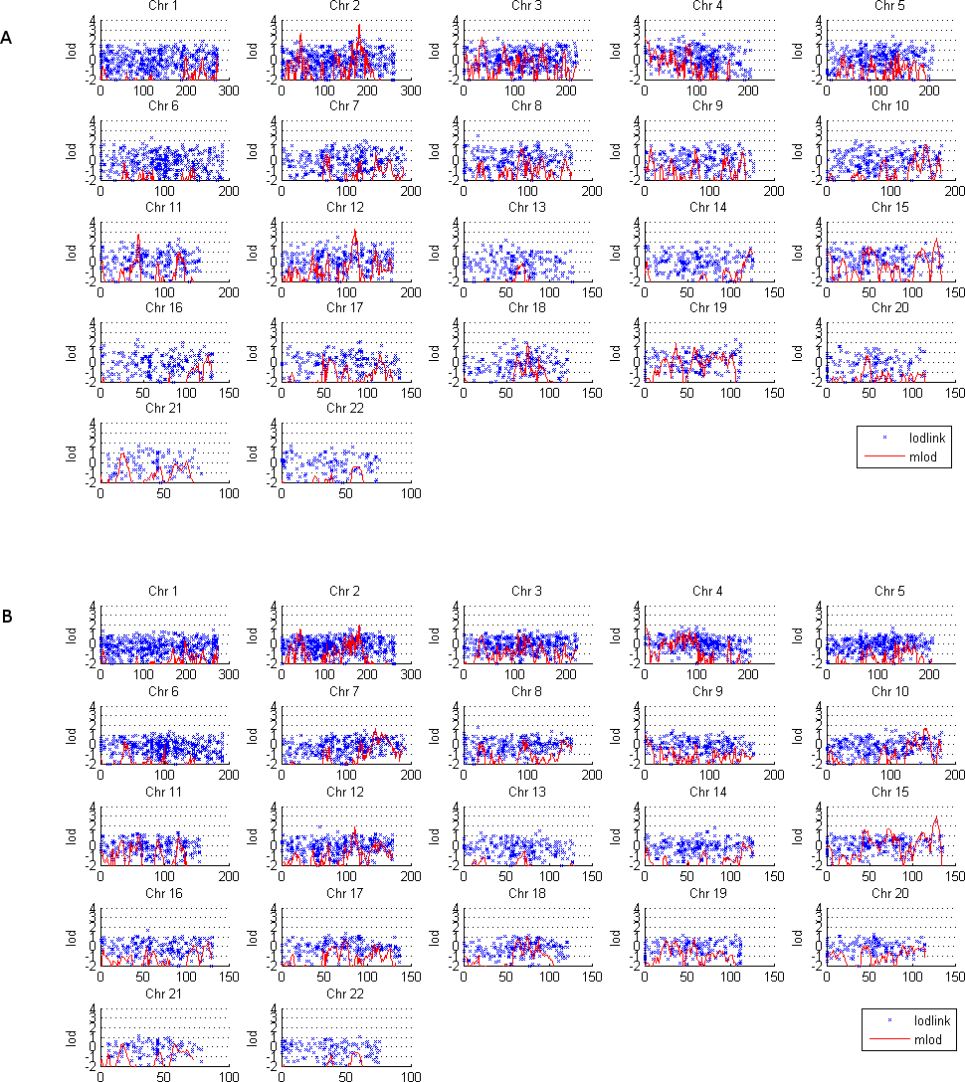
Model‐based linkage under the recessive model (model 2 in Table 1). (A) The 42 pedigrees. (B) The 18 female‐affected pedigrees. The red line is multipoint linkage by MLOD, the blue points are for single‐marker linkage by LODLINK. The *X*‐axis is the genetic position in cM, the *Y*‐axis is the lod.

**Figure 2:**
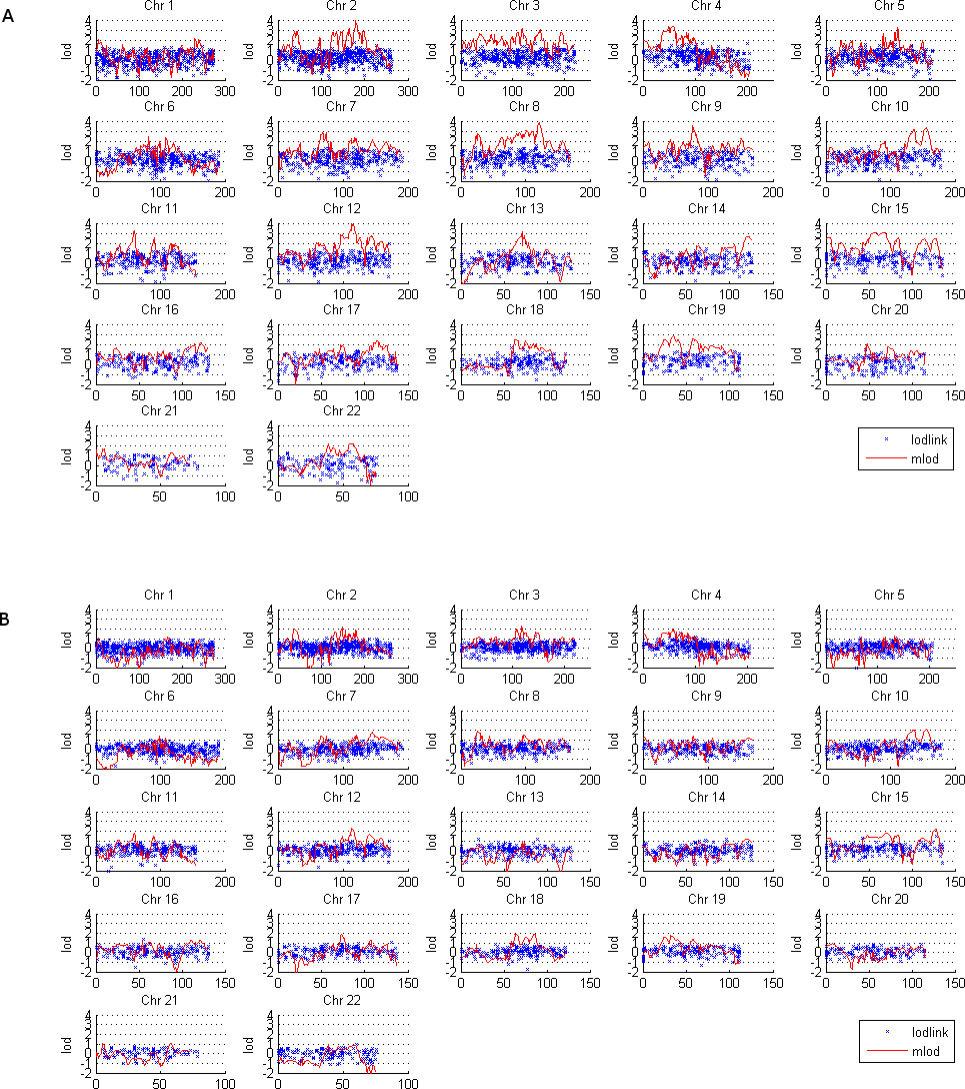
Model‐based linkage under the dominant model (model 1 in Table 1). (A) The 42 pedigrees. (B) The 18 female affected pedigrees. The red line is multipoint linkage by MLOD, the blue points are for single‐marker linkage by LODLINK. The *X*‐axis is the genetic position in cM, the *Y*‐axis is the lod.

Using the recessive model, there were some linkage (lod > 3) or suggestive linkage (lod > 2) regions by single‐marker or multipoint linkage analysis (Fig. 1A). Using the dominant model, there was no suggestive linkage by single‐marker linkage analysis. However, multipoint linkage analysis found many suggestive linkage regions (Fig. 2A, Table S2), and the dominant model led to an overall higher lod profile: the average lod under the dominant model was three lods higher than that under the recessive model. Therefore, only those regions under this model with lod > 3 are discussed here.

Under the recessive model, a wide, consistent linkage region was identified by both single‐marker and multipoint linkage analyses on chromosome 2q31 (174–190 cM), within which three SNPs have lod > 2 by single‐marker analysis (Table 2). This region was also supported by a multipoint linkage under the dominant model (lod > 4). A linkage region on chromosome 12 (110–120 cM) was identified by multipoint linkage under both dominant and recessive models, with lods of 3.9 and 3.3, respectively, at 113 cM (Figs. 1A and 2A, Table 2); we did not find any single‐marker linkage in this region.

The linkage region on chromosome 4 (48–59 cM) was identified by multipoint linkage under the dominant model (lod = 3.2), and a SNP with single‐marker linkage was identified in this region under the recessive model (lod = 2.0). Another region on this chromosome, at 73.2 cM, was identified by single marker linkage under the recessive model. A region on chromosome 8 at 99–125 cM was identified by model‐based multipoint linkage analyses under the dominant model (lod = 3.8 at 121 cM).

##### Model‐free linkage analysis

Single‐marker model‐free linkage in the 42 pedigrees identified one SNP having permutation *P* value < 0.0012 (or −log_10_(*P*) > 2.92), which corresponds to lod > 2 (Table S2). However, this SNP identified by model‐free linkage is not consistent with any other linkage analyses we performed.

#### Linkage analysis of the 18 female‐affected pedigrees

In the 18 female‐affected pedigrees, the only region that was consistently identified to have suggestive linkage by two analyses is on chromosome 15q26, identified by both the single‐marker and multipoint linkage analyses under the recessive model, with lods of 2.45 and 2.98, respectively, at 128.8 cM (Table 3). This region was also identified in the 42 pedigrees by multipoint linkage under the recessive model (Table S2), but the linkage signal in the 18 pedigrees is 0.6 lods higher.

**Table 3:** Linkage (LODLINK, MLOD, SIBPAL) and association (ASSOC) results identified using 18 female‐affected pedigrees with evidence of suggestive linkage or association

Chr	Position (cM)	Model‐based linkage								Model‐free linkage	Association
		Recessive						Dominant			
		LODLINK (lod > 2)			MLOD (lod > 2)			LODLINK (lod > 2)	MLOD (lod > 3)	SIBPAL (−log_10_(*P*) > 2.92)	ASSOC (*P* < 0.01)
		Position	SNP	Lod	Position	SNP	Lod	Lod	Lod	−log_10_(*P*)	*P*‐value
15	**128–130 (15q26)**	**128.83**	**rs2045112**	**2.445**	**128.83**	**rs2045112**	**2.979**	≤1.49	≤2.41	≤0.93	≥0.57
15					**130.14**	**rs7183000**	**2.309**				

Note: the regions or SNPs in bold are those identified by at least two linkage analyses. Regions or SNPs with suggestive linkage (lod > 2, or −log_10_(*P* > 2.92) or association (unadjusted *P* < 0.01) are reported; but, for multipoint linkage under the dominant model, only those with lod > 3 are reported because this model produces an overall higher average lod than the other linkage results. If no suggestive signal in a region is identified by an analysis, the largest signal by the analysis in the region is reported.

#### Estimating the proportion of linked pedigrees using Merlin

We also performed multipoint model‐based linkage using MERLIN (Abecasis et al. 2002), in order to estimate the proportion of linked pedigrees in our dataset. MERLIN gave linkage results similar to MLOD, estimating the proportion of linked pedigrees to be 88% at the linkage region on chromosome 2 in the 42 pedigrees, but only 74%, in the 18 female‐affected pedigrees. The proportion of linked pedigrees at the chromosome 15 region was 72% in the 42 pedigrees, but increased to 100% in the 18 female‐affected pedigrees.

### Association analysis

Not surprisingly, there were no SNPs reaching genome‐wide association significance by the association tests, using either the 42 pedigrees or the 18 female affected pedigrees (Figures S1 and S2). However, association tests with the 42 pedigrees identified 4 SNPs that showed some possible evidence of association in the linkage regions on chromosomes 2 (two SNPs in 2q31 had *P* ≤ 0.007), 4 (a SNP at 72.8 cM had *P* = 0.008), and 8 (a SNP with *P* = 0.002) (Table 2), although none of them would be significant when adjusting for multiple testing. No association was identified in the 18 female‐affected pedigrees at the linkage region identified by these pedigrees on chromosome 15.

### Summary of linkage and association analyses

In the 42 pedigrees, the linkage regions consistently identified by two or more linkage analyses are on chromosomes 2q31, 4p14, and 12q23. The wide linkage region on chromosome 2q31 (174–190 cM) was identified by multiple linkage analyses (single‐marker and multipoint linkage analyses under recessive and dominant models), and two SNPs in this region also showed suggestive association. The linkage region on chromosome 12q23 (110–120 cM) was identified by multipoint linkage under both dominant and recessive models. The region on 4p14 (48–59 cM) was identified by multipoint linkage under the dominant model, but with a single SNP linkage under the recessive model. Furthermore, chromosome 4q (72–73 cM) and 8q22 (99–125 cM) were identified in the 42 pedigrees by one of the linkage analyses and showed some evidence of association.

In the 18 female‐affected pedigrees, the only region identified by two or more linkage analyses was on chromosome 15q26 under the recessive model. This region was also identified in the 42 pedigrees, but the linkage was stronger in the subset of 18 pedigrees.

## Discussion

In this study, we performed extensive linkage analyses on Barrett's esophagus (BE) and its associated adenocarcinomas on 42 multiplex pedigrees and a subset of them, 18 female‐affected pedigrees. The best fitting inhertiance models were dominant or recessive, depending on the penetrance model we used and whether it included a polygenic component. By model‐based linkage analyses under the dominant or recessive models, regions on chromosomes 2q, 4p, and 12q were identified to have consistent linkage by at least two linkage analyses in the 42 pedigrees, and a narrow region on chromosome 15 was identified by model‐based linkage analyses under the recessive model in the 18 female‐affected pedigrees. Some other regions or SNPs on chromosome 4q and 8q were also identified by linkage and association analyses. All these linkage regions or positions are candidates for further study or verification and potential genetic testing in BE/EAC families.

It could be that the 42 pedigrees are not genetically different from the 18 female affected pedigrees and only appear to be so by chance. However, the proportion of linked pedigrees at the two linkage regions on chromosome 2 and chromosome 8 estimated by MERLIN suggests that, for the chromosome 2 linkage region, the 18 female‐affected pedigrees are more genetically heterogeneous than the whole set of 42 pedigrees. On the other hand, for the linkage on chromosome 15, the 18 female‐affected pedigrees are more genetically homogeneous than the 42 pedigrees.

In the previous segregation analysis, we found that BE was transmitted in a dominant mode by fitting the FPMM model (Sun et al. 2010). In this study, we also found that when we fit the FPMM model, using either the 1000 pedigrees or the 42 genotyped pedigrees, the best fitting model is a dominant model. But when we fit an MLM model without assuming residual associations between family members, the best fitting model is a recessive one. Using the appropriate genetic model in model‐based linkage analysis will increase the power to detect linkage. Our linkage results suggest our data comprise a combination of heterogeneous BE pedigrees, which would explain why one cannot clearly distinguish the mode of inheritance when assuming a single‐locus model.

There are some reported associations located in the linkage regions that we have identified. McElholm et al. (2010) studied IGF Axis Polymorphisms and reported SNPs in three genes (*IGF1*,* IGF1R*, and *GHR*) to be associated with BE, EAC, or reflux esophagitis. The associated SNPs in two of the genes, *IGF1* and *IGF1R,* are near the linkage peaks we have identified. The associated SNP rs6214 in *IGF1* that was reported by McElholm et al. is located in our linkage region on chromosome 12q23, 685 kb from the peak SNP rs3205421; but in another association study of BE, in a cohort of 1852 cases and 5172 controls, rs6214 did not reach genome‐wide significance (Palles et al. 2015). The associated SNPs (rs2715425 and rs4966044) in *IGF1R* are located in the linkage region on chromosome 15, 840 kb from the peak SNP rs2045112. Moreover, Orloff et al. (2011) reported linkage on 8q21 (rs3097418) and association on 8q22, which are both located in our linkage regions. The associated SNP rs3098233 they found on 8q22, in the *CTHRC1* gene, is 66 kb from the SNP rs2131858 in our linkage region (Table 2). Furthermore, Palles et al. (2015) reported two SNPs associated with BE by meta‐analysis. One is located on chromosome 2p24 (rs3072) near the first linkage peak we identified under the recessive model (Fig. 1A, Table S2), 320 kb from SNP rs952275, which has a lod of 2.44. The other one, (rs2701108) on chromosome 12, is 13 Mb from the peak of linkage we identified at 12q23; it is within the linkage region, with a lod of 1.55 at that location under the dominant model. These authors also detected a SNP in their discovery phase, rs10083033, which is 3.79 Mb (4 cM) from the linkage peak we found on 12q23. These reported associations support the evidence from our linkage findings. Furthermore, although there is no reported association located in the linkage region on chromosome 2q31 that we identified by multiple linkage analyses, prior studies have reported somatic mutations in genes mapping to this region in Barrett's adenocarcinomas (Walch et al. 2000; El‐Rifai et al. 2001; Bandla et al. 2012). In addition, in the Genotype‐Tissue Expression (GTEx) database (GTEx Consortium, 2015), we found that rs711814 in the chromosome 2 linkage region is a significant cis‐eQTL (*P* = 0.0000031) that regulates the KIAA1715 expressed in esophagus mucosa, and the peak SNP rs3205421 in the chromosome 12 region is a significant cis‐eQTL (*P* = 2 × 10^−13^) that regulates the GNPTAB gene expressed in esophagus muscularis (http://www.gtexportal.org/home/eqtls/). These reported functional effects further support our findings.

In this study, we genotyped all individuals with blood samples available. This is not an ideal study with a sufficiently large sample size to come up with results that can stand on their own. Nevertheless, the corroborative results already found in recent genome‐wide association studies demonstrate that thorough linkage analyses, even on non‐ideal data, can help focus the search for causal genes. On the reasonable assumption that it is genetically more homogeneous, we specifically studied linkage in the subset of 18 female‐affected pedigrees. It is always possible that females have some gender‐specific protective mechanisms from developing Barrett's esophagus, but the definitive reason for such a gender difference is unclear. An ideal linkage study would include more covariates, such as BMI and gastroesophageal reflux symptoms, easily done with the software we used. If, for example, evidence for linkage changes when BMI is included as a covariate, a genome‐wide association study of this obesity‐related disease would locate the risk variants by addressing the impact of BMI on the association(Schaid et al. 2003). Moreover, a denser genotyping array could have enabled us to identify more signals or provide a more precise location for the linked regions and association signals.

We anticipate that further studies with dense SNPs could refine our linkages and associations and verify our finding on chromosome 2q31, as well as in the other regions on chromosomes 4p, 4q, and 8q. Note that, given a linkage signal, the significance level of a verification association study would not need to reach a genome‐wide significance level. For the linkage region on chromosome 2, for example, which is about 18 cM long (i.e., a fraction of about 18/3000 of the whole genome), if we assume that 5 × 10^−8^ is the appropriate level for genome‐wide significance, a *P*‐value of 5 × 10^−8^ × 3000/18, or about 10^−5^, would be sufficient to allow for the multiple testing to validate our findings. Hence, we recommend the results of genome‐wide association studies be reevaluated in one of two ways. First, focusing on the limited number of linkage regions reported here, we could calculate the equivalent number of independent SNPs in those regions (Galwey 2009) to determine an appropriate P‐value and reexamine previous association study results. Second, investigate most of the linkage regions we have suggested where no associations have yet been found, in a new set of association data, but again with a much smaller multiple‐testing burden.

The rapidly rising incidence of EAC over the past four decades is undoubtedly related to an uncharacterized environmental factor. We propose that this change in the environmental factor is interacting with an underlying complex genetic susceptibility, which is contributing to this rising incidence of BE and EAC, and the genes involved will be easier to find if the linkage results we report here are taken into account.

## Conflict of Interest

None declared.

## Supporting information


**Data S1**. Description of the permutation test.
**Figure S1**. Minimum P values of association at each SNP on testing additive, dominant, and recessive effects respectively by the likelihood ratio test using 42 pedigrees, adjusting for sex and founder status.
**Figure S2**. Minimum P values of association at each SNP on testing additive, dominant, and recessive effects, respectively, by the likelihood ratio test using 18 female‐affected pedigrees, adjusting for sex and founder status.
**Table S1**. Penetrance functions of the four segregation models in Table 1.
**Table S2**. Summary of linkage (LODLINK, MLOD, SIBPAL) and association analyses (ASSOC) using all 42 pedigrees at the positions with suggestive linkage or association by any of the analyses.
**Table S3**. Summary of linkage (LODLINK, MLOD, SIBPAL) and association analyses (ASSOC) using 18 female affected pedigrees at the positions with suggestive linkage or association by any of the analyses.
