# Supplementary material for: Linkage and related analyses of Barrett's esophagus and its associated adenocarcinomas
Source: Mol Genet Genomic Med. 2016 Mar 14;4(4):407–19. doi: 10.1002/mgg3.211 (PMC4947860; doi:10.1002/mgg3.211)
Supplement: Supplementary file 1 — Data S1. Description of the permutation test. Figure S1. Minimum P values of association at each SNP on testing additive, dominant, and recessive effects respectively by the likelihood ratio test using 42 pedigrees, adjusting for sex and founder status. Figure S2. Minimum P values of association at each SNP on testing additive, dominant, and recessive effects, respectively, by the likelihood ratio test using 18 female‐affected pedigrees, adjusting for sex and founder status. Table S1. Penetrance functions of the four segregation models in Table 1. Table S2. Summary of linkage (LODLINK, MLOD, SIBPAL) and association analyses (ASSOC) using all 42 pedigrees at the positions with suggestive linkage or association by any of the analyses. Table S3. Summary of linkage (LODLINK, MLOD, SIBPAL) and association analyses (ASSOC) using 18 female affected pedigrees at the positions with suggestive linkage or association by any of the analyses. [file MGG3-4-407-s001.docx]

**Supplementary materials**

**Description of the permutation test**

The permutation test for significance of linkage is performed by shuffling within bins the sibpair IBD estimates obtained from GENIBD $(\hat{\pi}=f_{2}+0.5f_{1}$, where $f_{2}$ and $f_{1}$ are respectively the probabilities, given all the marker informaiton available in the pedigree, of the sib pair sharing 2 and 1 allele identical by descent) on the assumption that within bins they are approximately exchangeable. Each bin must be large enough to contain more than one IBD estimate, but small enough for them to be approximately exchangeble. The bins are therefore chosen to be the same size (plus or minus 1) and such that the total variance of an IBD estimate (variance within bins plus variance among bins) is minimized.

Consider a one-way ANOVA with unequal-size classes. Suppose *y_ij_* is the *j*-th observation in the *i*-th class, *n* is the total sample size, *n* = $\sum_{i=1}^{c} n_{i}$, $\bar{y_{i}}=\frac{1}{n_{i}}\sum_{j=1}^{n_{i}} y_{ij}$, $\bar{y}=\frac{1}{n}\sum_{i=1}^{c} \sum_{j=1}^{n_{i}} y_{ij}$. We then have the following ANOVA table, where SS is the sum of squares, corrected for the mean, MS is the mean square, df is degrees of freedom.

| Source | SS | df | MS |
| --- | --- | --- | --- |
| Among classes | $\sum_{i} {\bar{y_{i}}}^{2}-n\bar{y}^{2}$ | c-1 | M_A_ = $\frac{{SS}_{A}}{c-1}$ |
| Within class | $\sum_{i} \sum_{j} {y_{ij}}^{2}-\sum_{i} n_{i}{\bar{y_{i}}}^{2}$ | n-c | M_W_ = $\frac{{SS}_{W}}{n-c}$ |
| Total | $\sum_{i} \sum_{j} {y_{ij}}^{2}-n\bar{y}^{2}$ | n-1 |  |

The variance of *y_ij_*, $\sigma_{A}^{2}+\sigma_{w}^{2}$, can be estimated by $\frac{M_{A}-M_{W}}{k}+M_{W}$, where $k=\frac{1}{c}(n-\frac{1}{n}\sum_{i} n_{i}^{2})$. We thus identify classes with bins and $y_{ij}$ as the *j*-th IBD estimate in the *i*-*th* bin. For successive values of *c,* we form c bins such that every *y_j_* is in a bin and the bin sizes differ by no more than 1, and then estimate $\sigma_{A}^{2}+\sigma_{w}^{2}$*.* We stop as soon as the estimate increases, and use the bin size at the previous step as the optimal *c* (the one that miniimizes $\sigma_{A}^{2}+\sigma_{w}^{2}$).

Using this permutation algorithm, compared to the permutation test currently in SIBPAL, in 88% of the cases the P-value became less significant, in 12% of the cases they became more significant. It is anticipated that version 6.4 of S.A.G.E. will include this permutation test.


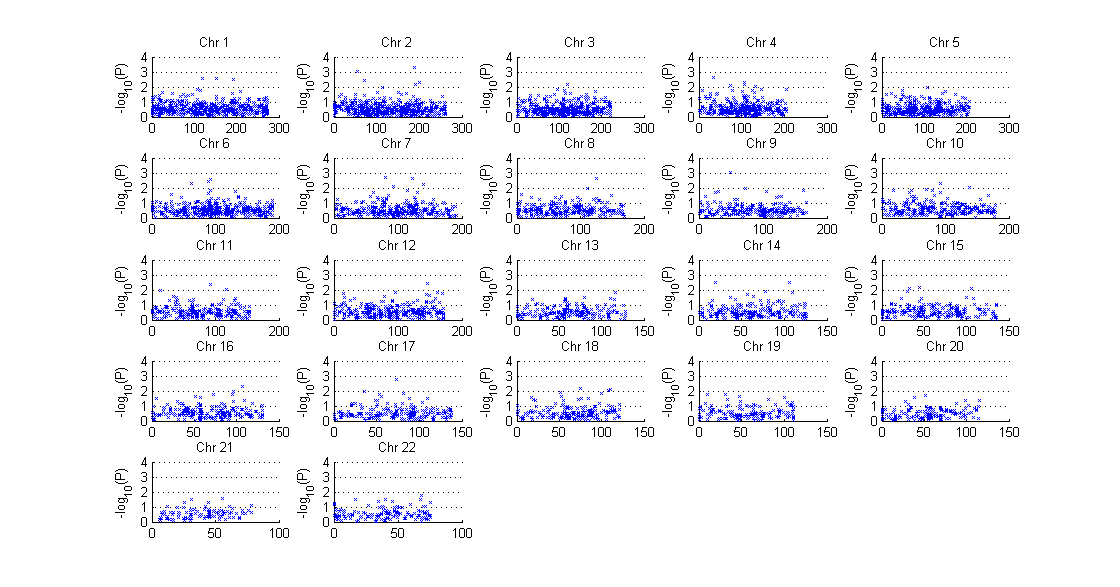


Supplementary FIG. S1. Minimum P values of association at each SNP on testing additive, dominant, and recessive effects respectively by the likelihood ratio test using 42 pedigrees, adjusting for sex and founder status.


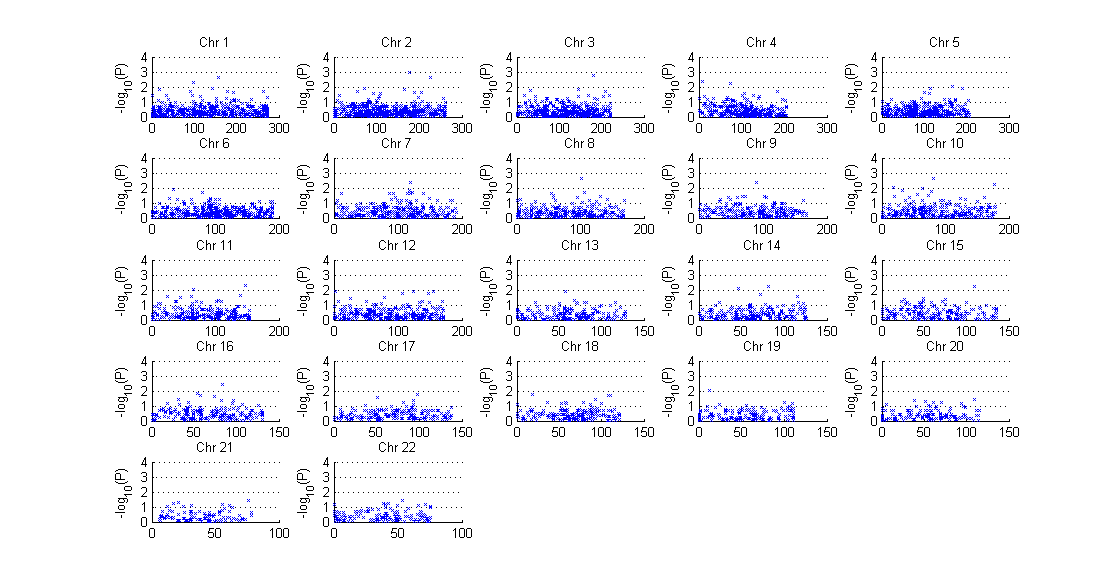


Supplementary FIG. S2. Minimum P values of association at each SNP on testing additive, dominant, and recessive effects, respectively, by the likelihood ratio test using 18 female affected pedigrees, adjusting for sex and founder status.

Supplementary table S1. Penetrance functions of the four segregation models in Table 1

|  | 42 pedigrees | | | | 1000 pedigrees | | | |
| --- | --- | --- | --- | --- | --- | --- | --- | --- |
|  | 1. Dominant | | 2. Recessive | | 3. Dominant | | 4. Recessive | |
|  | AA or AB | BB | AA | AB or BB | AA or AB | BB | AA | AB or BB |
| male nonfounder | 0.25 | 9.9×10^-15^ | 0.72 | 2.6×10^-15^ | 0.26 | 0.01 | 0.66 | 0.02 |
| female nonfounder | 0.03 | 8.0×10^-16^ | 0.24 | 3.3×10^-16^ | 0.08 | 2.7×10^-3^ | 0.31 | 4.7×10^-3^ |
| male founder | 0.01 | 4.0×10^-16^ | 0.15 | 1.8×10^-16^ | 0.07 | 2.2×10^-3^ | 0.26 | 3.7×10^-3^ |
| female founder | 0.001 | 3.2×10^-17^ | 0.02 | 2.2×10^-17^ | 0.02 | 5.5×10^-4^ | 0.07 | 8.6×10^-4^ |
| freq | q_A_ = 0.05 | | q_A_ = 0.21 | | q_A_ = 0.005 | | q_A_ = 0.07 | |

Note: Susceptibility or penetrance = e^β+sex+founder^/(1+ e^β+sex+founder^), where β, sex and founder are the estimated coefficients for a given genotype, female sex, and founder, respectively; they are the parameters of susceptibility on the logit scale.

Supplementary Table S2 Summary of linkage (LODLINK, MLOD, SIBPAL) and association analyses (ASSOC) using all 42 pedigrees at the positions with suggestive linkage or association by any of the analyses

|  |  | Model-based linkage | | | | | | | | | | Model-free linkage | | | Association | | |
| --- | --- | --- | --- | --- | --- | --- | --- | --- | --- | --- | --- | --- | --- | --- | --- | --- | --- |
|  |  | Recessive | | | | | | Dominant | | | |  | | |  | | |
|  |  | LODLINK (lod>2) | | | MLOD (lod>2) | | | LODLINK (lod>2) | MLOD (lod>3) | | | SIBPAL (-log_10_(P) > 2.92) | | | ASSOC (P < .01) | | |
| Chr | Position (cM) | Position | SNP | lod | Position | SNP | lod |  | Position | SNP | lod | Position | SNP | -log_10_(P) | Position | SNP | P-value |
| 1 | 118 |  |  |  |  |  |  |  |  |  |  |  |  |  | 118.94 | rs1573065 | 0.0026 |
|  | 153 |  |  |  |  |  |  |  |  |  |  |  |  |  | 153.17 | rs857819 | 0.0029 |
|  | 192 |  |  |  |  |  |  |  |  |  |  |  |  |  | 192.37 | rs2039759 | 0.0031 |
| 2 | 42-46  (2p24) |  |  |  | 42.87 | rs952275 | 2.44 |  |  |  |  |  |  |  |  |  |  |
|  |  |  |  |  | 44.28 | rs1463979 | 2.59 |  |  |  |  |  |  |  |  |  |  |
|  |  |  |  |  | 44.5 | rs1997325 | 2.49 |  |  |  |  |  |  |  |  |  |  |
|  |  |  |  |  | 44.71 | rs2001795 | 2.60 |  |  |  |  |  |  |  |  |  |  |
|  |  |  |  |  | 46.13 | rs925229 | 2.20 |  |  |  |  |  |  |  |  |  |  |
|  | 55 |  |  |  |  |  |  |  |  |  |  |  |  |  | 55.92 | rs927087 | 0.0009 |
|  | 70 |  |  |  |  |  |  |  |  |  |  |  |  |  | 70.71 | rs10427402 | 0.0040 |
|  | 163 |  |  |  |  |  |  |  | 163.4 | rs1482308 | 3.09 |  |  |  |  |  |  |
|  |  |  |  |  |  |  |  |  | 163.87 | rs11682209 | 3.12 |  |  |  |  |  |  |
|  | **174 -190**  **(2q31)** | **174.27** | **rs2032965** | **2.15** | **178.55** | **rs11078** | **2.08** |  | **177.35** | **rs17664** | **3.13** |  |  |  |  |  |  |
|  |  | **180.30** | **rs935866** | **2.30** | **179.18** | **rs907956** | **2.39** |  | **178.55** | **rs11078** | **3.49** |  |  |  |  |  |  |
|  |  |  |  |  | **179.48** | **rs3813817** | **3.23** |  | **179.18** | **rs907956** | **3.64** |  |  |  |  |  |  |
|  |  |  |  |  | **179.76** | **rs2033866** | **3.47** |  | **179.48** | **rs3813817** | **3.93** |  |  |  |  |  |  |
|  |  |  |  |  | **180.3** | **rs935866** | **3.60** |  | **179.76** | **rs2033866** | **4.02** |  |  |  |  |  |  |
|  |  |  |  |  | **181.55** | **rs711814** | **2.6058** |  | **180.3** | **rs935866** | **3.97** |  |  |  |  |  |  |
|  |  |  |  |  |  |  |  |  | **181.55** | **rs711814** | **3.61** |  |  |  |  |  |  |
|  |  |  |  |  |  |  |  |  | **183.55** | **rs1438048** | **3.43** |  |  |  | **189.74** | **rs1569135** | **0.0005** |
|  |  |  |  |  |  |  |  |  | **183.87** | **rs1400826** | **3.41** |  |  |  | **190.70** | **rs11894667** | **0.0071** |
|  | 201 |  |  |  |  |  |  |  |  |  |  |  |  |  | 201.47 | rs7602656 | 0.0048 |
|  | 225 | 225.10 | rs721357 | 2.03 |  |  |  |  |  |  |  |  |  |  |  |  |  |
| 3 | 65 | 65.33 | rs1995137 | 2.12 |  |  |  |  |  |  |  |  |  |  |  |  |  |
|  | 118 |  |  |  |  |  |  |  |  |  |  |  |  |  | 118.62 | rs1447713 | 0.0069 |
| 4 | 26 | 26.38 | rs758879 | 2.05 |  |  |  |  |  |  |  |  |  |  |  |  |  |
|  | 34 |  |  |  |  |  |  |  |  |  |  |  |  |  | 34.78 | rs964095 | 0.0022 |
|  | 48-59  (4p14) |  |  |  |  |  |  |  |  |  |  |  |  |  |  |  |  |
|  |  |  |  |  |  |  |  |  | 49.04 | rs1553923 | 3.27 |  |  |  |  |  |  |
|  |  |  |  |  |  |  |  |  | 49.65 | rs1392056 | 3.31 |  |  |  |  |  |  |
|  |  |  |  |  |  |  |  |  | 51.18 | rs902659 | 3.14 |  |  |  |  |  |  |
|  |  | **53.25** | **rs13134283** | **2.02** |  |  |  |  | **53.25** | **rs13134283** | **3.16** |  |  |  |  |  |  |
|  |  |  |  |  |  |  |  |  | 56.7 | rs1115259 | 3.38 |  |  |  |  |  |  |
|  |  |  |  |  |  |  |  |  | 58.87 | rs278973 | 3.34 |  |  |  |  |  |  |
|  |  |  |  |  |  |  |  |  | 59.46 | rs2035383 | 3.37 |  |  |  |  |  |  |
|  | 65 | 65.43 | rs225160 | 2.13 |  |  |  |  |  |  |  |  |  |  |  |  |  |
|  | 72-73 (4q13) | 73.21 | rs1390351 | 2.31 |  |  |  |  |  |  |  |  |  |  | 72.78 | rs11727819 | 0.0077 |
|  | 105 |  |  |  |  |  |  |  |  |  |  |  |  |  | 105.83 | rs716556 | 0.0051 |
|  | 107 |  |  |  |  |  |  |  |  |  |  |  |  |  | 107.44 | rs747559 | 0.0071 |
|  | 112 | 112.56 | rs2035415 | 2.59 |  |  |  |  |  |  |  |  |  |  |  |  |  |
|  | 131 |  |  |  |  |  |  |  |  |  |  |  |  |  | 131.79 | rs2055664 | 0.0086 |
| 5 | 128 | 128.83 | rs1439564 | 2.37 |  |  |  |  |  |  |  |  |  |  |  |  |  |
| 6 | 60 |  |  |  |  |  |  |  |  |  |  |  |  |  | 61.75 | rs629832 | 0.0050 |
|  | 80 | 80.52 | rs977382 | 2.23 |  |  |  |  |  |  |  |  |  |  |  |  |  |
|  | 88 |  |  |  |  |  |  |  |  |  |  |  |  |  | 88.76 | rs1486039 | 0.0037 |
|  | 92 |  |  |  |  |  |  |  |  |  |  |  |  |  | 92.25 | rs4111785 | 0.0028 |
|  | 144 |  |  |  |  |  |  |  |  |  |  | 144.8 | rs2027195 | 4 |  |  |  |
| 7 | 79 |  |  |  |  |  |  |  |  |  |  |  |  |  | 79.95 | rs1874243 | 0.0021 |
|  | 92 |  |  |  |  |  |  |  |  |  |  |  |  |  | 92.43 | rs1468242 | 0.0074 |
|  | 121 |  |  |  |  |  |  |  |  |  |  |  |  |  | 121.97 | rs1123065 | 0.0024 |
|  | 139 |  |  |  |  |  |  |  |  |  |  |  |  |  | 139.84 | rs1582634 | 0.0057 |
| 8 | 23 | 23.00 | rs1435277 | 2.40 |  |  |  |  |  |  |  |  |  |  |  |  |  |
|  | 100-125  (8q22) |  |  |  |  |  |  |  | 100.14 | rs11785 | 3.01 |  |  |  |  |  |  |
|  |  |  |  |  |  |  |  |  | 101.69 | rs2245832 | 3.09 |  |  |  |  |  |  |
|  |  |  |  |  |  |  |  |  | 102.23 | rs714046 | 3.08 |  |  |  |  |  |  |
|  |  |  |  |  |  |  |  |  | 111.38 | rs2131858 | 2.94 |  |  |  |  |  |  |
|  |  |  |  |  |  |  |  |  | 118.86 | rs1628373 | 3.43 |  |  |  |  |  |  |
|  |  |  |  |  |  |  |  |  | 119.53 | rs769322 | 3.71 |  |  |  |  |  |  |
|  |  |  |  |  |  |  |  |  | 121.52 | rs2034844 | 3.83 |  |  |  |  |  |  |
|  |  |  |  |  |  |  |  |  | 122.14 | rs1433396 | 3.74 |  |  |  |  |  |  |
|  |  |  |  |  |  |  |  |  | 122.36 | rs1021898 | 3.73 |  |  |  |  |  |  |
|  |  |  |  |  |  |  |  |  | 124.76 | rs755649 | 3.23 |  |  |  | 125.32 | rs748856 | 0.0024 |
| 9 | 49 |  |  |  |  |  |  |  |  |  |  |  |  |  | 49.59 | rs666478 | 0.0007 |
|  | 75-77 |  |  |  |  |  |  |  | 75.6 | rs2839899 | 3.33 |  |  |  |  |  |  |
|  |  |  |  |  |  |  |  |  | 75.81 | rs1887773 | 3.46 |  |  |  |  |  |  |
|  |  |  |  |  |  |  |  |  | 76.48 | rs722642 | 3.34 |  |  |  |  |  |  |
|  |  |  |  |  |  |  |  |  | 76.88 | rs735914 | 3.33 |  |  |  |  |  |  |
|  |  |  |  |  |  |  |  |  | 77.13 | rs1417080 | 3.37 |  |  |  |  |  |  |
| 10 | 92 |  |  |  |  |  |  |  |  |  |  |  |  |  | 92.03 | rs877783 | 0.0052 |
|  | 137 |  |  |  |  |  |  |  |  |  |  |  |  |  | 137.22 | rs758367 | 0.0090 |
| 11 | 13 |  |  |  |  |  |  |  |  |  |  |  |  |  | 13.53 | rs2280726 | 0.0089 |
|  | 58-60 |  |  |  | 58.78 | rs975104 | 2.68 |  |  |  |  |  |  |  |  |  |  |
|  |  |  |  |  | 59.46 | rs1518816 | 2.77 |  |  |  |  |  |  |  |  |  |  |
|  |  |  |  |  | 59.9 | rs4755782 | 2.65 |  |  |  |  |  |  |  |  |  |  |
|  | 92 |  |  |  |  |  |  |  |  |  |  |  |  |  | 91.97 | rs1880116 | 0.0044 |
|  | 118 |  |  |  |  |  |  |  |  |  |  |  |  |  | 117.91 | rs609177 | 0.0087 |
|  | 121 | 121.07 | rs547345 | 2.29 |  |  |  |  |  |  |  |  |  |  |  |  |  |
| 12 | 72 | 72.10 | rs774033 | 2.01 |  |  |  |  |  |  |  |  |  |  |  |  |  |
|  | 87 | 87.20 | rs1400142 | 2.42 |  |  |  |  |  |  |  |  |  |  |  |  |  |
|  | **110-120**  **(12q23)** |  |  |  | **111.16** | **rs1492254** | **2.55** |  | **111.16** | **rs1492254** | **3.35** |  |  |  |  |  |  |
|  |  |  |  |  | **112** | **rs35723** | **2.57** |  | **112** | **rs35723** | **3.31** |  |  |  |  |  |  |
|  |  |  |  |  | **112.36** | **rs11609259** | **2.55** |  | **112.36** | **rs11609259** | **3.25** |  |  |  |  |  |  |
|  |  |  |  |  | **113.35** | **rs3205421** | **3.26** |  | **113.35** | **rs3205421** | **3.94** |  |  |  |  |  |  |
|  |  |  |  |  | **115.83** | **rs703618** | **2.33** |  | **114.73** | **rs6539055** | **3.73** |  |  |  |  |  |  |
|  |  |  |  |  | **116.17** | **rs1000295** | **2.31** |  | **115.83** | **rs703618** | **3.83** |  |  |  |  |  |  |
|  |  |  |  |  |  |  |  |  | **116.17** | **rs1000295** | **3.80** |  |  |  |  |  |  |
|  |  |  |  |  |  |  |  |  | **117.17** | **rs746035** | **3.68** |  |  |  |  |  |  |
|  |  |  |  |  |  |  |  |  | **119.05** | **rs1862032** | **3.09** |  |  |  |  |  |  |
|  | 145 |  |  |  |  |  |  |  |  |  |  |  |  |  | 145.65 | rs2197777 | 0.0037 |
| 13 | 50 | 49.97 | rs625052 | 2.16 |  |  |  |  |  |  |  |  |  |  |  |  |  |
| 14 | 19 |  |  |  |  |  |  |  |  |  |  |  |  |  | 19.46 | rs1957528 | 0.0034 |
|  | 106 |  |  |  |  |  |  |  |  |  |  |  |  |  | 106.64 | rs10131135 | 0.0031 |
| 15 | 32 |  |  |  |  |  |  |  |  |  |  |  |  |  | 32.51 | rs1551650 | 0.0079 |
|  | 44 |  |  |  |  |  |  |  |  |  |  |  |  |  | 44.03 | rs1426932 | 0.0071 |
|  | 105 |  |  |  |  |  |  |  |  |  |  |  |  |  | 105.25 | rs1122702 | 0.0084 |
|  | 128 |  |  |  | 128.83 | rs2045112 | 2.30 |  |  |  |  |  |  |  |  |  |  |
| 16 | 42 | 42.30 | rs2031077 | 2.20 |  |  |  |  |  |  |  |  |  |  |  |  |  |
|  | 107 |  |  |  |  |  |  |  |  |  |  |  |  |  | 107.31 | rs2967305 | 0.0052 |
| 17 | 73 |  |  |  |  |  |  |  |  |  |  |  |  |  | 73.49 | rs1230101 | 0.0017 |
|  | 91 | 91.37 | rs12451501 | 2.00 |  |  |  |  |  |  |  |  |  |  |  |  |  |
| 18 | 59 | 59.09 | rs972900 | 2.24 |  |  |  |  |  |  |  |  |  |  |  |  |  |
|  | 75 |  |  |  |  |  |  |  |  |  |  |  |  |  | 75.58 | rs1145315 | 0.0066 |
|  | 108 |  |  |  |  |  |  |  |  |  |  |  |  |  | 108.45 | rs999647 | 0.0087 |
|  | 110 |  |  |  |  |  |  |  |  |  |  |  |  |  | 110.96 | rs1943193 | 0.0081 |
| 19 | 105 | 105.49 | rs7479 | 2.23 |  |  |  |  |  |  |  |  |  |  |  |  |  |

Note: the regions or SNPs in bold are those identified by at least two linkage analyses. Regions or SNPs with suggestive linkage (lod > 2, or (-log_10_(P) > 2.92) or association (unadjusted P < .01) are reported, but, for multi-point linkage under the dominant model, those with lod > 3 are reported because it has an overall higher average lod than the other linkage results.

Supplementary Table S3. Summary of linkage (LODLINK, MLOD, SIBPAL) and association analyses (ASSOC) using 18 female affected pedigrees at the positions with suggestive linkage or association by any of the analyses

|  |  | Model-based linkage | | | | | | | | Model-free linkage | | | Association | | |
| --- | --- | --- | --- | --- | --- | --- | --- | --- | --- | --- | --- | --- | --- | --- | --- |
|  |  | Recessive | | | | | | Dominant | |  | | |  | | |
|  |  | LODLINK (lod>2) | | | MLOD (lod>2) | | | LODLINK (lod>2) | MLOD (lod>3) | SIBPAL (-log_10_(P) > 2.92) | | | ASSOC (P < .01) | | |
| Chr | Position (cM) | Position | SNP | lod | Position | SNP | lod | Position | Position | Position | SNP | -log_10_(P) | Position | SNP | P-value |
| 1 | 97 |  |  |  |  |  |  |  |  |  |  |  | 96.88 | rs2842040 | 0.0053 |
|  | 157 |  |  |  |  |  |  |  |  |  |  |  | 157.06 | rs570901 | 0.0022 |
| 2 | 176 |  |  |  |  |  |  |  |  |  |  |  | 176.02 | rs2032965 | 0.0011 |
|  | 227 |  |  |  |  |  |  |  |  |  |  |  | 226.85 | rs721357 | 0.0023 |
| 3 | 182 |  |  |  |  |  |  |  |  |  |  |  | 181.57 | rs1545866 | 0.0018 |
| 4 | 7 |  |  |  |  |  |  |  |  |  |  |  | 7.11 | rs934498 | 0.0040 |
|  | 73 |  |  |  |  |  |  |  |  |  |  |  | 72.78 | rs11727819 | 0.0058 |
| 5 | 167 |  |  |  |  |  |  |  |  |  |  |  | 167.32 | rs2069347 | 0.0090 |
| 7 | 120 |  |  |  |  |  |  |  |  |  |  |  | 119.59 | rs719530 | 0.0043 |
| 8 | 102 |  |  |  |  |  |  |  |  |  |  |  | 101.69 | rs2245832 | 0.0022 |
| 9 | 90 |  |  |  |  |  |  |  |  |  |  |  | 89.99 | rs7043803 | 0.0044 |
| 10 | 18 |  |  |  |  |  |  |  |  |  |  |  | 17.89 | rs713588 | 0.0088 |
|  | 81 |  |  |  |  |  |  |  |  |  |  |  | 80.61 | rs377859 | 0.0023 |
|  | 177 |  |  |  |  |  |  |  |  |  |  |  | 177.41 | rs12570033 | 0.0056 |
| 11 | 75 |  |  |  |  |  |  |  |  |  |  |  | 74.52 | rs4077638 | 0.0086 |
|  | 148 |  |  |  |  |  |  |  |  |  |  |  | 147.86 | rs2044727 | 0.0048 |
| 14 | 46 |  |  |  |  |  |  |  |  |  |  |  | 46.30 | rs1947393 | 0.0077 |
|  | 81 |  |  |  |  |  |  |  |  |  |  |  | 80.98 | rs2060596 | 0.0060 |
| 15 | 110 |  |  |  |  |  |  |  |  |  |  |  | 109.53 | rs7176568 | 0.0055 |
|  | **128-130** | **128.83** | **rs2045112** | **2.445** | **128.83** | **rs2045112** | **2.979** |  |  |  |  |  |  |  |  |
|  |  |  |  |  | **130.14** | **rs7183000** | **2.309** |  |  |  |  |  |  |  |  |
| 16 | 83 |  |  |  |  |  |  |  |  |  |  |  | 83.07 | rs973200 | 0.0037 |
| 19 | 12 |  |  |  |  |  |  |  |  |  |  |  | 12.18 | rs1715093 | 0.0087 |

Note: the regions or SNPs in bold are those identified by at least two linkage analyses. Regions or SNPs with suggestive linkage (lod > 2, or (-log_10_(P) > 2.92) or association (unadjusted P < .01) are reported, but, for multi-point linkage under the dominant model, those with lod > 3 are reported because it has an overall higher average lod than the other linkage results.
